# Supplementary material for: A Critical View of the Application of the APEX Software (Aqueous Photochemistry of Environmentally-Occurring Xenobiotics) to Predict Photoreaction Kinetics in Surface Freshwaters
Source: Molecules. 2019 Dec 18;25(1):9. doi: 10.3390/molecules25010009 (PMC7017383; doi:10.3390/molecules25010009)
Supplement: Supplementary file 1 [file molecules-25-00009-s001.zip › 3.2.4_gcc-4.4.0.zip!/3.2.4_gcc-4.4.0/3.2.4_gcc-4.4.0/doc/octave/html/interpreter/Recursion.html]

Recursion - Untitled


Previous: Call by Value,
Up: Calling Functions


---

#### 8.2.2 Recursion

With some restrictions^1^, recursive function calls are allowed. A
recursive function is one which calls itself, either directly or
indirectly. For example, here is an inefficient^2^ way to compute the factorial of a given integer:

```
     function retval = fact (n)
       if (n > 0)
         retval = n * fact (n-1);
       else
         retval = 1;
       endif
     endfunction
```

This function is recursive because it calls itself directly. It
eventually terminates because each time it calls itself, it uses an
argument that is one less than was used for the previous call. Once the
argument is no longer greater than zero, it does not call itself, and
the recursion ends.

The built-in variable `max_recursion_depth` specifies a limit to
the recursion depth and prevents Octave from recursing infinitely.

— Built-in Function: val = **max\_recursion\_depth** ()  
— Built-in Function: old\_val = **max\_recursion\_depth** (new\_val)  
> Query or set the internal limit on the number of times a function may
> be called recursively. If the limit is exceeded, an error message is
> printed and control returns to the top level.

---

#### Footnotes

[1] Some of Octave's functions are
implemented in terms of functions that cannot be called recursively.
For example, the ODE solver `lsode` is ultimately implemented in a
Fortran subroutine that cannot be called recursively, so `lsode`
should not be called either directly or indirectly from within the
user-supplied function that `lsode` requires. Doing so will result
in an error.

[2] It would be
much better to use `prod (1:n)`, or `gamma (n+1)` instead,
after first checking to ensure that the value `n` is actually a
positive integer.

---
